# Surface-based functional metrics and auditory cortex characteristics in chronic tinnitus

**DOI:** 10.1016/j.heliyon.2022.e10989

**Published:** 2022-10-08

**Authors:** Xiaoyan Ma, Ningxuan Chen, Fangyuan Wang, Chi Zhang, Jing Dai, Haina Ding, Chaogan Yan, Weidong Shen, Shiming Yang

**Affiliations:** a The First Affiliated Hospital of Xi'an, Jiaotong University, Shanxi, China; b Medical School of Chinese PLA, Beijing, China; c Department of Otolaryngology Head and Neck Surgery, Chinese PLA General Hospital, Beijing, China; d National Clinical Research Center for Otolaryngologic Diseases, Beijing, China; e Key Lab of Hearing Science, Ministry of Education, Beijing, China; f Beijing Key Lab of Hearing Impairment Prevention and Treatment, Beijing, China; g CAS Key Laboratory of Behavioral Science, Institute of Psychology, Beijing, China; h Magnetic Resonance Imaging Research Center, Institute of Psychology, Chinese Academy of Sciences, Beijing, China; i International Big-Data Center for Depression Research, Chinese Academy of Sciences, Beijing, China; j Department of Psychology, University of Chinese Academy of Sciences, Beijing, China; k Center for Cognitive Science of Language, Beijing Language and Culture University, Beijing, China; l Department of Child and Adolescent Psychiatry, Hassenfeld Children's Hospital at NYU Langone, New York, NY, USA

**Keywords:** Tinnitus, Secondary auditory cortex, Resting-state fMRI

## Abstract

Abnormal auditory cortex (AC) neuronal activity is thought to be a primary cause of the auditory disturbances perceived by individuals suffering from tinnitus. The present study was designed to test that possibility by evaluating auditory cortical characteristics (volume, curvature, surface area, thickness) and surface-based functional metrics in chronic tinnitus patients. In total, 63 chronic tinnitus patients and 36 age-, sex- and education level-matched healthy control (HC) patients were enrolled in this study. Hearing levels in these two groups were comparable, and following magnetic resonance imaging (MRI) of these individuals, the DPABISurf software was used to compute cerebral cortex curvature, thickness, and surface area as well as surface-based functional metrics. The Tinnitus Handicap Inventory (THI), Tinnitus Handicap Questionary (THQ), and Visual Analogue Scales (VAS) were used to gauge participant tinnitus severity, while correlation analyses were conducted to evaluate associations between these different analyzed parameters. A significant increase in the regional homogeneity (ReHo) of the right secondary AC was detected in the tinnitus group relative to the HC group. There were also significant reductions in the cortical volume and surface area of the right secondary AC in the tinnitus group relative to the HC group (all P < 0.05). In addition, significant negative correlations between tinnitus pitch and the cortical area and volume of the right secondary AC were observed in the tinnitus group.

## 1 Introduction

Individuals suffering from tinnitus perceive sounds in the absence of corresponding auditory stimuli, and may exhibit corresponding cortical changes. An estimated 2–3% of the overall population is thought to suffer from tinnitus (Bhatt et al., 2016), and these rates rise with age such that 14.3% of the general population in the seventh decade of life are afflicted by this condition (Shargorodsky et al., 2010). Tinnitus is not thought to solely arise due to ear dysfunction, and commonly coincides with peripheral hearing loss of varying severity (Stohler et al., 2019). Such hearing loss generally precedes tinnitus onset, particularly for higher frequency hearing not assessed during traditional clinical audiological testing (Eggermont 2015). In prior studies, chronic tinnitus has been linked to a range of neuropathological alterations including increases in spontaneous neuronal activity (Noreña and Eggermont 2003), neural synchrony, tonotopic map reorganization (Noreña and Farley 2013), aberrant consciousness gating (Zeng 2013), and auditory and non-auditory structure-based network coupling (Lockwood et al., 1998; Kaltenbach et al., 2005; Chen et al., 2017). The neurological basis for tinnitus, however, remains to be clarified.

The resting human brain represents only 2% of total body mass yet consumes 20% of the body's energy, most of which is used to support ongoing neuronal signaling. Task-related increases in neuronal metabolism are generally small (<5%) when compared with this large resting energy consumption (Fox and Raichle 2007). Because of its subjective nature, tinnitus may be uniquely suited to being studied using a resting-state functional connectivity paradigm (Husain and Schmidt 2014). As tinnitus is a chronic condition, it can be effectively evaluated via resting-state functional magnetic resonance imaging (fMRI), which has revealed a link between tinnitus pathogenesis and both functional (Mirz et al., 2000; Smits et al., 2007; Schlee et al., 2009; Vanneste et al., 2010; De Ridder, Vanneste et al., 2011; van der Loo et al., 2011; Vanneste et al., 2011) and structural changes in the brain (MacDonald et al., 2000; Mühlau et al., 2006; Landgrebe et al., 2009; Husain et al., 2011; Leaver et al., 2011). Such resting-state fMRI analyses have also revealed that tinnitus itself is governed by both auditory and non-auditory functional neural networks. In tinnitus patients, abnormalities are most often detected in the auditory cortex (AC) region of the brain, with fMRI analyses frequently revealing hyperactivity in this region (Kim et al., 2012; Maudoux et al., 2012; Maudoux, Lefebvre et al. 2012a, Maudoux et al., 2012b). Increased steady-state metabolic activity has also been reported in the AC upon positron emission tomography (PET) examination (Wang et al., 2001; Langguth et al., 2006; Schecklmann et al., 2013a). Owing to discrepancies in sample sizes, fMRI analysis approaches, and the underlying hearing status of tinnitus patients included in these prior studies, however, it is possible that inconsistencies may have been introduced into these analyses. As such, in the present study, we specifically focused on tinnitus patients with normal hearing in an effort to control for heterogeneity. Given that the AC is critically important in tinnitus, we focused on this as a region of interest (ROI). MRI analyses generally exhibit spatial localization just 35% of that for the best surface-based methods as determined based upon peak areal probabilities and captured area fraction for maximum probability maps (Coalson et al., 2018). Owing to significant heterogeneity, it can be challenging to reconcile results from studies utilizing a standard Voxel-Based Morphometry (VBM) approach (Mühlau et al., 2006; Landgrebe et al., 2009; Husain et al., 2011; Leaver et al., 2011; Boyen et al., 2013; Schecklmann et al., 2013b). Adjamian et al. identified 17 morphometric studie comparing patients with tinnitus to control participants without tinnitus (Adjamian et al., 2014). Reported tinnitus-related changes occurred in both auditory and non-auditory areas, with these changes putatively being associated with the acoustic and emotional aspects of tinnitus, respectively. However, reported findings are often contradictory, suggesting that inconsistencies among these studies may be attributable to VBM measurement-related methodological limitations (Adjamian et al., 2014). Prior MRI studies have also primarily focused on parameters including cortical volume, area, curvature, and thickness, while not analyzing other surface-based functional metrics that have the potential to offer more insight into cortical characteristics that may underlie tinnitus pathology (Yoo et al., 2016; Neuschwander et al., 2019). We therefore employed an innovative surface-based morphometry (SBM) analytical approach in this study to more fully explore tinnitus structural and functional signatures (Leaver et al., 2012).

For the present analysis, we began by examining multiple surface-based functional metrics (Amplitude of Low-Frequency Fluctuation, fraction Amplitude of Low-Frequency Fluctuation, Degree centrality, and ReHo) and cortical characteristics (thickness, surface area, and curvature) in chronic tinnitus patients with normal hearing to better explore tinnitus pathophysiology. We also began by using the early AC and secondary AC as ROIs generated with the multi-modal MRI scans from the Human Connectome Project (HCP) (Glasser et al., 2016). We hypothesized that patients with tinnitus with normal hearing were likely to exhibit hyperactive neural patterns and structural changes in the AC and that these abnormalities may be correlated with characteristics of tinnitus including pitch, duration, and/or severity.

## 2 Methods

### 2.1 Subjects

**Figure 1:**
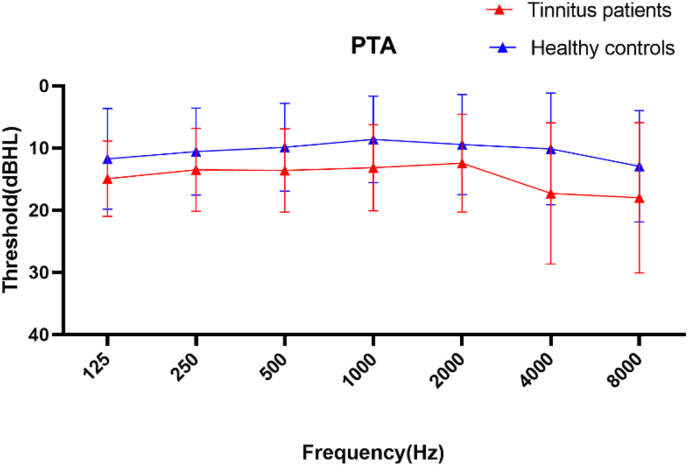
Average hearing thresholds of tinnitus patients and healthy controls. Data are means ± SD.

In total, we enrolled 99 right-handed individuals with 8 or more years of education who were recruited through community health screens and newspaper advertisements between September 2019 and September 2020. Of these participants, 63 suffered from chronic tinnitus, while the remaining 36 were healthy controls (HCs). Participants were found to be unaffected by depression or anxiety as determined using the Self-Rating Depression Scale (SDS) and Self-Rating Anxiety Scale (SAS) (overall score <50). Patients were not eligible to participate in this study if they had a history of hyperacusis, with subjects exhibiting a score >28.4 being considered to be hyperacusic (Khalfa et al., 2012). Similarly, subjects were excluded if they had a history of pulsatile tinnitus, Meniere's disease, heavy smoking, alcoholism, stroke, brain injury, neurodegenerative conditions, epilepsy, major depression, other neurological or psychiatric conditions, major illnesses (including cancer, anemia, and thyroid disorders), or MRI contraindications (Internal cardiac pacemakers; Implantable cardiac defibrillators; Cochlear implants; Neurostimulators; Bone growth stimulators; Implantable electronic drug-infusion pumps; Shrapnel in vital locations; Ferromagnetic eye prostheses; Weight >136 kg; Aneurysm clips; Certain heart valves; Metallic vascular access ports; Orthopedic implants of less than 3 months' duration) (Goh et al., 1999). Patients were eligible for inclusion in the chronic tinnitus group if they were (1) ≥ 18 years old; (2) had suffered from tinnitus for ≥6 months; and (3) exhibited normal hearing as defined by hearing thresholds <25 dB HL at any of 7 measured audiometric frequencies between 125 Hz and 8 kHz with normal tympanograms. Individuals were eligible to be enrolled in the HC group if they were (1) ≥ 18 years old; (2) free of tinnitus; and (3) exhibited normal hearing as defined by the above criteria. Of the enrolled tinnitus patients, one was excluded due to excessive head movement during MRI scanning. Of the remaining 62 tinnitus patients, 39 presented with bilateral tinnitus, 15 had predominantly left-sided tinnitus, and 9 had predominantly right-sided tinnitus. Tinnitus-relates distress and tinnitus severity were analyzed using the Tinnitus Handicap Inventory (THI) (Newman et al., 1996), Tinnitus Handicap Questionary (THQ), and Visual Analogue Scales (VAS). Pure tone audiometry (PTA) was utilized to assess hearing thresholds. No significant differences in these thresholds were detected between patient groups across the measured range (Figure 1 and Table 1). The psychoacoustic characteristics of the tinnitus spectrum (pitch and loudness) were assessed. Pitch matching was conducted by presenting patients with two tones and asking them to select the tone that was closer to the perceived sound and with matches being made monaurally and repeated three times prior to designating a match. Choices were repeated until a match was made. Loudness matching was conducted by presenting the patient with a matched frequency tone that was just below threshold and then gradually increasing the loudness of this tone in 5 dB HL increments until a match was made (Newman et al., 2014).

**Table 1:** Tinnitus patient and healthy control hearing threshold.

Frequency (Hz)	Tinnitus group PTA threshold (dBHL)	Control group PTA threshold (dBHL)	P value
125	14.91 ± 6.01	11.74 ± 8.05	0.08
250	13.5 ± 6.63	11.56 ± 6.95	0.13
500	13.58 ± 6.65	10.86 ± 7.02	0.09
1000	13.17 ± 6.69	9.62 ± 6.93	0.07
2000	12.41 ± 7.80	9.74 ± 8.03	0.11
4000	16.29 ± 11.32	12.14 ± 8.95	0.06
8000	17.00 ± 12.04	13.91 ± 8.91	0.06

Data are means ± SD.

Ethics Committee Of Chinese PLA General Hospital (Beijing, China) had approved this study and Written informed consent was obtained from all subjects enrolled in this study.

### 2.2 MRI scanning

A GE 3T MR750 scanner with an 8-channel receiver array head coil was used to conduct all imaging. Foam padding and earplugs were used to limit head motion and scan-related noise. Patients were directed to remain still with their eyes closed and to avoid thinking about anything in particular while the scan was occurring. Blood oxygenation level-dependent (BOLD) signal was captured with the following echoplanar imaging (EPI) sequence:Repetition time (TR) = 2000 ms, echo time (TE) = 30 ms, flip angle = 90°, 3 mm isotropic voxels. The duration of the resting-state period scan was 8 min and 4 s. In addition, a T1-weighted (T1w) structural image was acquired (FSPGR 3D sequence: TR = 8.2 ms, TE = 3.2 ms, flip angle = 8°).

### 2.3 Analysis of cortical characteristics

**Figure 2:**
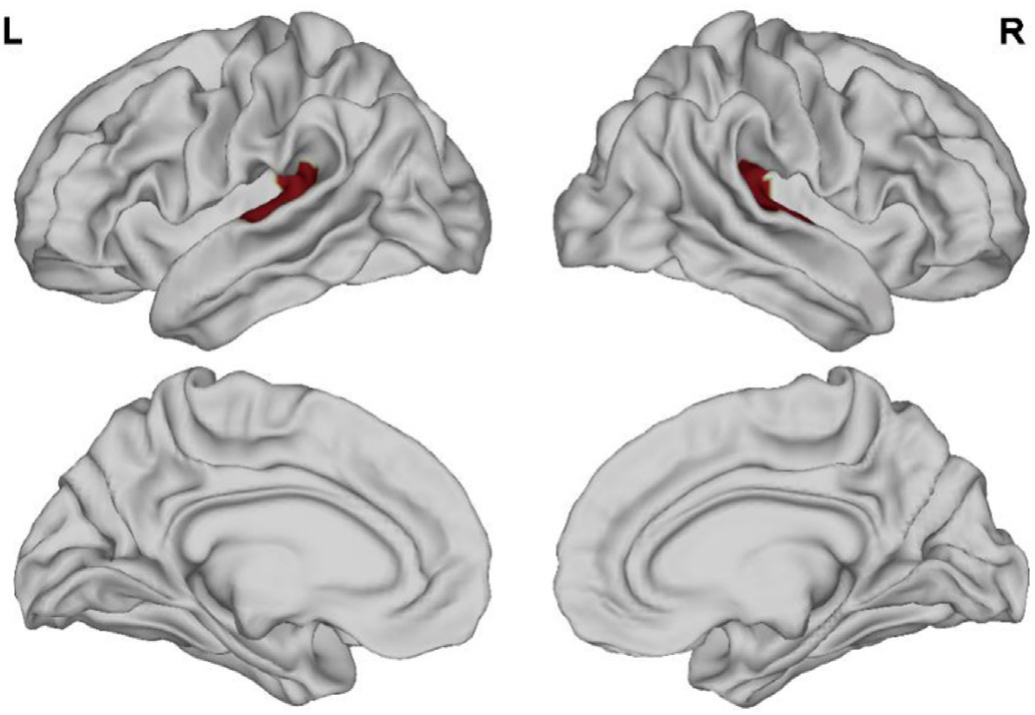
Primary auditory cortex mask.

**Figure 3:**
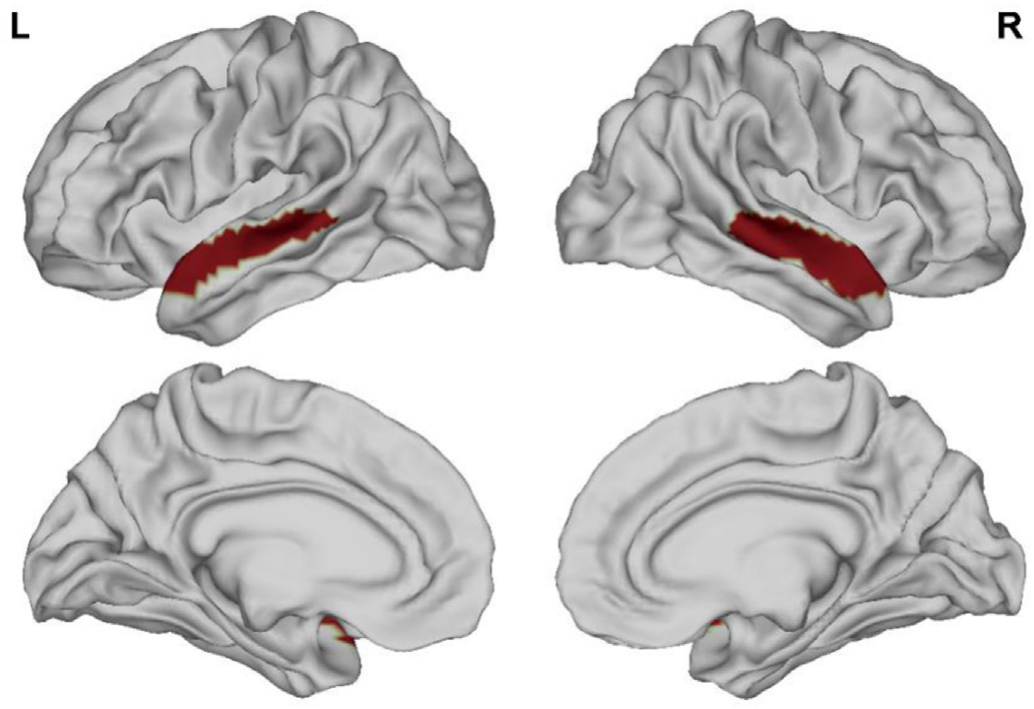
Secondary auditory cortex mask.

T1w MRI images were analyzed with the DPABISurf_V1.2 software (http://rfmri.org/DPABISurf) in an automated manner to calculate cortical thickness, surface area, volume, and curvature (Yan and Zang 2010). Recon-all (FreeSurfer 6.0.) was used to reconstruct brain surfaces (Dale et al., 1999), and the previously estimated brain mask was refined with a custom variation of the method used to reconcile ANTs-derived and FreeSurfer-derived segmentation of the cortical gray-matter of Mindboggle (Klein et al., 2017). A nonlinear regression analysis conducted with antsRegistration (ANTs 2.2.0) was used to achieve spatial normalization to the ICBM 152 Nonlinear Asymmetrical template v. 2009c based upon the T1w and template. Based upon cortical atlas labels, the DPABISurf software yielded parameters including mean cortical thickness, surface area, volume, and curvature. A threshold-free cluster enhancement (TFCE) approach was used to correct for multiple comparisons, and a significance threshold of P < 0.025 was used. Four different surface-based functional metrics (ALFF, fALFF, DC, and ReHo) were selected to represent different functional aspects. ALFF corresponds to the mean amplitude of low-frequency fluctuations (0.01–0.1 Hz) based on the fast Fourier transformation in the time course for each voxel (Zang et al., 2007). fALFF is a normalized ALFF that is determined based on total power within the low-frequency range (0.01–0.1 Hz) divided by the overall power of the full frequency range of the same voxel (Zou et al., 2008). DC is the number or sum of significant connections weights for each voxel. ReHo represents the homogeneity of a time course for a given voxel relative to that of the time courses of the 26 nearest neighboring voxels, calculated based upon Kendall's coefficient of concordance (KCC) (Zang et al., 2004). ROI seeds for the left and right AC were generated based upon multimodal MRI scans from the Human Connectome Project (HCP) (Glasser et al., 2016), with the early AC (Figure 2) and secondary AC (Figure 3) having been selected as ROIs.

## 3 Statistical analyses and results

### 3.1 Statistical analysis

To test for possible anatomical or functional differences between tinnitus and HC participant cohorts, vertex-wise group comparisons were made through permutation-based statistical analyses with 5000 permutations, with hearing thresholds, age, sex, education, and the head motion serving as covariates and with a statistical threshold of P < 0.025. A TFCE approach was used to control for multiple comparisons. SPSS 20.0 (IBM SPSS Inc, IL, USA) was used to analyze tinnitus-related characteristics. Correlation analyses were used to evaluate associations between tinnitus characteristics, hyperactive hearing-related neural patterns (pitch, duration, and severity, including VAS, THI, and THQ), and surface-based functional parameters, cortical surface area, curvature, thickness, and volume in participant groups. Demographic data were compared between groups via Student's t-tests and Fisher's exact test. PTA thresholds in these two groups were compared through the use of two-sample t-tests. All analyses were two-tailed with P < 0.05 as the threshold of significance, and data are given as means ± standard deviation (SD).

### 3.2 Demographic and clinical data

The demographic and clinical data pertaining to patients in the tinnitus and HC groups are shown in Table 1 and Table 2. There were no significant differences in age, sex, education status, or hearing thresholds when comparing these groups.

**Table 2:** Tinnitus patient and healthy control characteristics.

	Tinnitus Patients (n = 63)	Healthy Controls (n = 36)	p Value
Age (years)	40.8 ± 13.2	45.2 ± 11.9	0.174
Gender (male:female)	40:23	21:15	0.777
Education (years)	16.7 ± 2.64	17.1 ± 2.24	0.633
Tinnitus duration (months)	42.6 ± 41.4	-	-
THI total score	41.4 ± 19.7	-	-
THQ	56.1 ± 13.9	-	-
VAS	7.31 ± 2.20	-	-

Data are means ± SD. THI, Tinnitus Handicap Inventory, THQ, Tinnitus Handicap Questionnaire. VAS, Visual Analogue Scales.

### 3.3 Cortical characteristic differences

**Figure 4:**
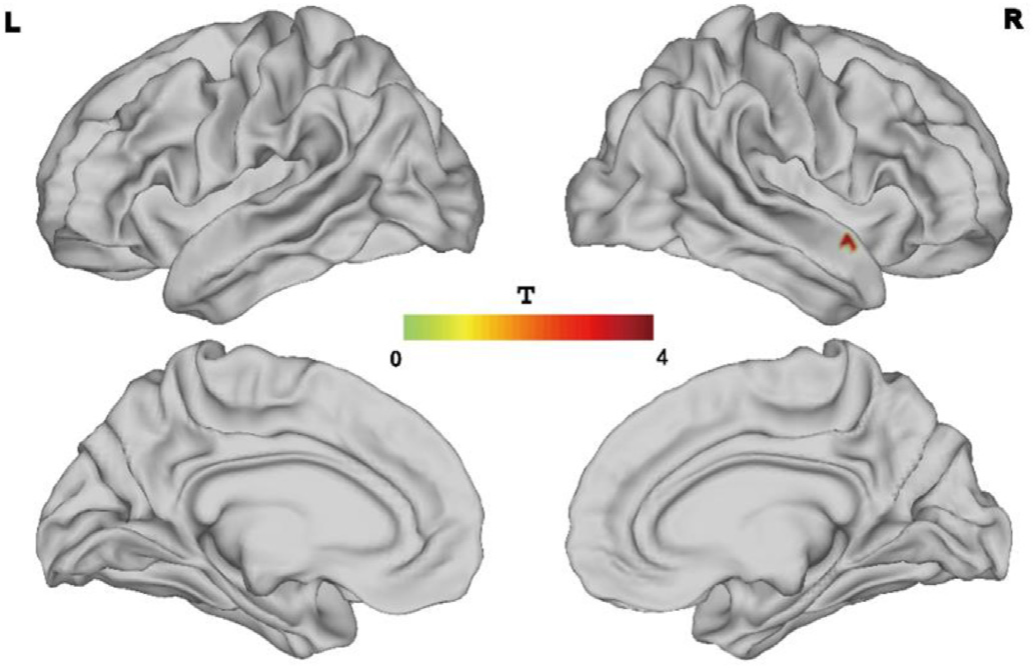
Significant increases were found in regional homogeneity (ReHo) in the right secondary auditory cortex were detected for patients in the tinnitus group (P < 0.025 for all, corrected by TFCE).

Relative to individuals in the HC group, In which hearing thresholds, average cortical volume and surface area has been included as group variance, we observed significant increases in regional homogeneity (ReHo) in the right secondary auditory cortex among patients in the tinnitus group (Figure 4). The cortical volume and surface area of the right secondary auditory cortex were also significantly decreased in tinnitus patients relative to HCs (all P < 0.025, corrected by TFCE). Cortical curvature and thickness did not differ significantly between these two groups.

### 3.4 Correlation analyses

**Figure 5:**
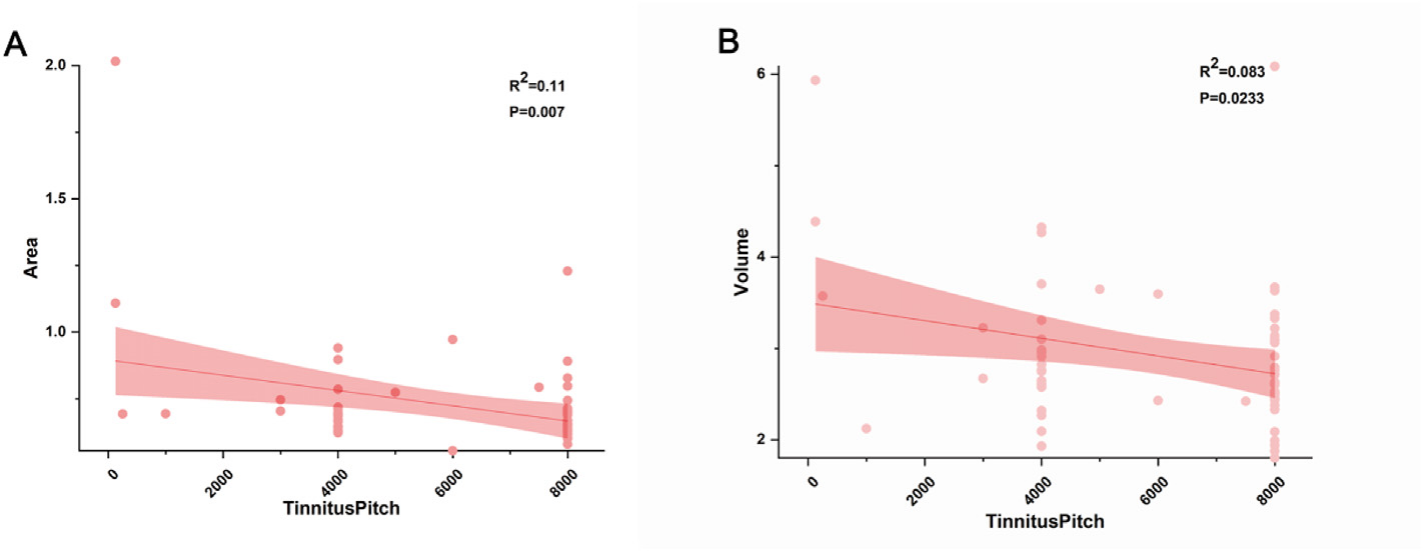
A. The cortical area of the right secondary auditory cortex was significantly negatively correlated with tinnitus pitch. B. The cortical volume of the right secondary auditory cortex was significantly negatively correlated with tinnitus pitch.

Significant negative correlations between tinnitus pitch and the cortical volume and area of the right secondary auditory cortex were observed among patients in the tinnitus group (Figure 5A and 5B). Consistently, the cortical area of the right secondary auditory cortex was significantly negatively correlated with tinnitus pitch (R^2^ = 0.11, P = 0.007), as was the cortical volume of the right secondary auditory cortex (R^2^ = 0.083, P = 0.0223). There was no significant correlations between VAS/THI/THQ and the cortical characteristic differences were observed among patients in the tinnitus group (see Table 3).

**Table 3:** Correlation test between cortical characteristic and Tinnitus characteristics.

TC	R and P value of correlation test between cortical characteristic and TC		
	ReHo and TC	cortical volume and TC	surface area and TC
THI	R^2^ = 0.004, P = 0.850	R^2^ = 0.025, P = 0.222	R^2^ = 0.025, P = 0.220
VAS	R^2^ = 0.012, P = 0.398	R^2^ = 0.064, P = 0.060	R^2^ = 0.158, P = 0.903
THQ	R^2^ = 0.020, P = 0.270	R^2^ = 0.165, P = 0.390	R^2^ = 0.027, P = 0.061
Tinnits duration	R^2^ = 0.007, P = 0.513	R^2^ = 0.002, P = 0.904	R^2^ = 0.176, P = 0.716
Tinnitus pitch	R^2^ = 0.11, P = 0.007∗	R^2^ = 0.083, P = 0.022∗	R^2^ = 0.238, P = 0.070

TC: tinnitus characteristics; ReHo: ReHo in the right secondary auditory cortex; cortical volume: cortical volume of the right secondary auditory cortex; surface area: surface area of the right secondary auditory cortex; THI, Tinnitus Handicap Inventory, THQ, Tinnitus Handicap Questionnaire. VAS, Visual Analogue Scales. ∗:P < 0.05.

## 4 Discussion

Herein, we explored the importance of the auditory cortex in patients with tinnitus by analyzing surface-based functional metrics (ALFF, fALFF, DC, and ReHo), cortical thickness, surface area, degree of mean curvature, and gray matter volume. These analyses confirmed our hypothesis that tinnitus patients with normal hearing have abnormal changes in the secondary auditory cortex and that there is a correlation between tinnitus pitch and abnormal secondary auditory complex area and volume.

Tinnitus is a condition wherein affected individuals perceive a particular sound in the absence of any corresponding internal or external auditory stimulus. Tinnitus can impair patient quality of life, particularly when it contributes to poorer sleep quality or is complicated by anxiety and/or depression (Langguth 2011). A range of cochlear pathologies such as infection or acoustic trauma can precede tinnitus incidence, although in other cases it may be idiopathic in nature. At present, it is hypothesized that cochlear damage, even when too small to cause substantial hearing loss, can reduce input to the auditory network in the brain (Henry et al., 2014). In an effort to maintain homeostasis between network inputs and outputs, this is thought to result in the amplification of auditory inputs such that spontaneous neuronal firing at rest can result in the perception of a sound (Schaette and Kempter 2006; Yang et al., 2011). In line with this hypothesis, both humans and animals have been shown to exhibit AC hyperactivity in the context of tinnitus (Gu et al., 2010; Eggermont 2015). We similarly observed such AC hyperactivity in tinnitus patients with normal hearing when using surface-based functional metrics to ensure greater overall study accuracy.

The primary AC (HG) and secondary AC (PT) have previously been shown to be impacted by tinnitus (Mühlau et al., 2006; Boyen et al., 2013) and hearing loss (Mühlau et al., 2006; Boyen et al., 2013; Vanneste et al., 2015). Tinnitus, for example, was found to have a borderline impact on both the HG and PT, with some evidence of decreases in cortical thickness in the supratemporal gyrus (Schneider et al., 2009; Aldhafeeri et al., 2012; Allan et al., 2016). However, these results are not universal, and others have instead reported increased tinnitus-related cortical thickness of the AC (Husain et al., 2011; Boyen et al., 2013). Prior research has similarly shown hearing impairment to be linked with reductions in the thickness of the primary and secondary AC regions (Neuschwander et al., 2019). The inconsistencies between these past studies may be attributable to differences in sample size, baseline patient hearing, or fMRI analysis approaches. In an effort to minimize such heterogeneity in the present study, we specifically focused on chronic tinnitus patients exhibiting normal hearing, and we utilized SBM-based fMRI analytical methods. Through this approach, we detected significant decreases in the area and volume of the right secondary AC in tinnitus patients relative to HC patients.

The main structures that are involved in auditory stimulus processing, including the primary AC (Heschl's gyrus) and the secondary AC (Heschl's sulcus), can undergo reorganization such that they are associated with processing information related to visual, tactile, or sign language inputs (Petitto et al., 2000; Glick and Sharma 2017). While the primary AC appears normal in tinnitus patients who exhibit normal hearing, there are significant differences in their secondary AC structures. The ReHo of the right secondary AC was significantly increased in tinnitus patients, possibly due to the reductions in right secondary AC volume and cortical area. We also detected significant negative correlations between tinnitus pitch and the cortical area and volume of the right secondary AC in these patients (Figure 5). Real-time fMRI neurofeedback studies of the AC rely on the ability of participants to regulate their brain activity based upon feedback from a particular fMRI activity metric, and such regulation has been shown to be more pronounced in regions of the secondary AC (Emmert et al., 2017). This suggests that portions of the secondary AC are likely to be more sensitive to voluntary modulation relative to the primary AC (Carmen and Svihovec 1984; Puckett et al., 2007). In one animal study, tinnitus was speculated to arise as a consequence of the increased spontaneous firing rate of the secondary AC rather than the primary AC (Eggermont and Kenmochi 1998). Our data similarly support an important role for the secondary AC in chronic tinnitus patients with normal hearing, indicating that it may be a viable target for future neuromodulation efforts. In this study which included 26 subjects with single-sided tinnitus, there was 12 left tinnitus and 14 right tinnitus, howerer, all findings were lateralized to the right hemisphere. In line with Lanting's study which he found that the unilateral tinnitus (8 left-sided, 6 right sided) sound-evoked responses did not relate to the laterality of tinnitus. The lateralization for left- or right ear stimuli, as expressed in a lateralization index, was considerably smaller in subjects with tinnitus compared to that in controls reaching significance in the right primary auditory cortex (PAC) and the right inferior colliculus (IC) (Lanting et al., 2009).

There are certain limitations to this analysis. For one, while we took efforts to ensure consistency between the HC and tinnitus patient groups when selecting study subjects, these efforts significantly decreased the number of subjects in each group and may have limited the statistical power of our analyses. In addition, even when tinnitus patients exhibit normal PTA results, other in-depth analyses may reveal the presence of pathological hearing-related conditions that were overlooked in the present analysis. The precision of MRI analyses is also limited, and 7T-based MRI systems have the potential to improve the results of this study. Whether these sensitivity improvements, however, would be sufficient to detect additional subtle morphological changes in the cortical regions of interest remains to be determined.

## 5 Conclusion

In conclusion, we observed a significant increase in the ReHo of the right secondary auditory cortex in the tinnitus group relative to the HC group, potentially owing to differences in the cortical volume and surface area of the right secondary auditory cortex between these two cohorts, suggesting that the secondary auditory cortex plays a key role in regulating tinnitus. In addition, the area and volume of the right secondary auditory cortex were negatively correlated with tinnitus pitch, and as such this right secondary auditory cortex region may represent a viable target for neurofeedback or other treatments in tinnitus patients with normal hearing.

## Declarations

### Author contribution statement

Xiaoyan Ma: Analyzed and interpreted the data; Contributed reagents, materials, analysis tools or data; Wrote the paper.

Ningxuan Chen and Fangyuan Wang: Analyzed and interpreted the data; Wrote the paper.

Chaogan Yan, Weidong Shen and Shiming Yang: Conceived and designed the experiments.

Zhang Chi, Jing Dai and Haina Ding: Performed the experiments.

### Funding statement

Fangyuan Wang was supported by Beijing Nova Program [Z201100006820133].

Weidong Shen was supported by National Basic Research Program of China (973 Program) [2019YFC0121302 & 2019YFC0840707].

### Data availability statement

Data will be made available on request.

### Declaration of interests statement

The authors declare no conflict of interest.

### Additional information

The clinical trial described in this paper was registered at Ethics Committee for Chinese PLA General Hospital and Approval under the registration number S2021-558-01.
